# Federal health workforce policy in Australia and its implications: a descriptive policy document review

**DOI:** 10.5694/mja2.70021

**Published:** 2025-08-17

**Authors:** Stephanie M Topp, Thu Nguyen, Lana M Elliott

**Affiliations:** ^1^ James Cook University Townsville QLD; ^2^ Queensland University of Technology Brisbane QLD

**Keywords:** Health policy, Health personnel, Delivery of healthcare

## Abstract

**Objective:**

To identify which federal health workforce policies are current in Australia, and describe their mode, scope, and focus.

**Study design:**

Descriptive policy document review; categorisation according to the Howlett–Ramesh policy instrument framework.

**Setting:**

Health workforce policy documents available on the Australian Department of Health and Aged Care website, 1 June – 31 October 2024.

**Main outcome measures:**

Primary policy focus (specific health profession, population group or location); scope of policy (alignment with one or more strategic domains: supply, distribution, or performance), service sectors affected by policy, substantive mention of specific health professions; policy instrument types.

**Results:**

We included 121 policy documents in our analysis. By policy group, the number of documents was greatest for the rural health workforce (35), aged care (22), and Aboriginal and Torres Strait Islander health workforce (19); the numbers were lowest for pharmacy (three) and allied health (one), and none had public health or emergency care as their focus. Mixed policy instruments (multiple interest group programs, sub‐programs, incentives, grants) were more numerous (98 documents) than government‐led instruments (23 documents). Health workforce supply was a focus of 72 documents, performance of 57 documents, and distribution of 42 documents. Document nomenclature was inconsistent; 44 documents had policy labels that did not correspond to their content or purpose.

**Conclusion:**

We identified substantial fragmentation in Australian federal health workforce policy. The absence of a unified federal health workforce strategy exacerbates policy fragmentation, undermining coordinated workforce planning and equity. Adopting a consistent policy nomenclature and reducing imbalances in strategic focus are critical for effective health workforce reform. Our findings provide a baseline for analyses of policy processes and governance in Australian health workforce policymaking.

**The known**: Health workforce policies are critical for reducing workforce shortages and ensuring equitable access to care in Australia, but the federal policy landscape is poorly understood.

**The new**: Important gaps and inconsistencies in policy focus in 121 current federal policy documents related to the health workforce include limited attention to allied health and pharmacy, reliance on short term solutions, and inconsistent policy labelling.

**The implications**: A unified health workforce strategy and standardised policy categories are essential for improving the coordination and equity of health workforce planning, required for the long term resilience of the Australian health system.

Strong national health workforce policy is fundamental to a responsive and adaptable health system. By coordinating action and investment at the national level, federal government health workforce policy can set consistent national directions for workforce training, distribution, and regulation, establishing a foundation upon which state and territory systems can build local responses. This approach supports crisis adaptation, workforce management, and equitable access.^1^ This adaptability is particularly essential in a country like Australia, where policies must navigate the complexities of a shared labour market, with an interconnected workforce that spans federal and state jurisdictions, the public and private health sectors, and health‐relevant sectors such as disability and aged care.

The Australian health system is frequently lauded as being one of the best in the world, in part because of the capabilities of its health professionals.^2^ But as health care demands intensify, Australia faces a health workforce crisis.^3^ Even with a record 852 272 registered health practitioners (as at 30 June 2022), demand continues to surpass supply, opening significant gaps in both private and public sector health care.^4^ Position vacancy rates are high for nearly all health professions, and many health professionals now choose to work in private practice or part‐time.^5^ Shortages are widespread across the country, affecting medicine,^6^ dentistry,^7^ nursing,^8^ midwifery,^9^ and allied health,^10^ and are particularly intense in rural and remote locations.^11^ Meanwhile, long waiting times are increasingly typical in the primary and tertiary sectors; some practitioners and organisations cannot accept new patients, and many rural and remote communities do not have permanent health care providers.^12^ The slowly moving but intensifying crisis raises fundamental questions about the federal and jurisdictional policy frameworks that guide health workforce planning in Australia.

For two decades, successive reports have recommended a national workforce policy. The 2005 Productivity Commission report, *Australia’s health workforce*, highlighted the complex and fragmented nature of health workforce planning and recommended establishing an advisory health workforce council to evaluate and facilitate major workforce innovation at the national level.^13^ In its 2009 final report, *A healthier future for all Australians*, the National Health and Hospitals Reform Commission made 123 recommendations, including providing national leadership and system‐wide integration for the optimal use of resources and knowledge.^14^ And the Mason *Review of Australian government health workforce programs*, commissioned in 2013, reinforced the importance of coherent education and training pathways across all health professions to reduce distribution imbalances and service delivery gaps.^1^ These domestic recommendations are aligned with those of overseas assessments, such as the 2016 Organisation for Economic Co‐operation and Development report, *Health workforce policies in OECD countries*, which emphasised the importance of integrated health workforce policies for meeting systemic demands.^15^

Despite such recommendations, Australia has no national health workforce policy, nor a national coordinating body for health workforce policy and governance. Health Workforce Australia, established as part of the 2008 National Partnership Agreement on Hospital and Health Workforce Reform for this purpose, was disbanded in 2014 as part of a raft of federal budget measures for improving administrative efficiency.^16^, ^17^ Without a federal strategy or governance lead, it is difficult to ascertain which health workforce policies target which problems (eg, workforce shortages and equitable access), how they intersect, or the extent to which they set a consistent direction.

As a starting point for improving policy coordination and reform, we therefore undertook a systematic analysis in which we identified which federal health workforce policies are current, their strategic and professional focus, scope, and mode.

## Methods

The objectives of our descriptive policy review were to collate all available federal policy documents regarding the health workforce in Australia, and to categorise the policies by document type, health profession, policy authors, and health workforce policy domains. Our findings could provide a basis for critiques or analyses of these policies, but we did not undertake such analyses in this study.

A broad definition of policy includes formal and informal rules, guidelines, and instruments that shape decisions and actions in the public sphere. However, as our focus was on formal health workforce policy, we adopted the narrower definition of the formal and structural set of decisions, actions, and instruments used by governments and authoritative bodies to direct health workforce planning.^18^ As part of our analysis, we used an adapted version of Howlett and Ramesh’s policy instrument framework,^19^ which is widely used in public policy scholarship for categorising policy tools by level of government involvement (government‐led, voluntary, mixed/partnership) and the processes by which policy objectives are pursued.

### Data collection

The Australian Department of Health and Aged Care, as the primary author of national health workforce policy, was the primary data source. In the first phase, we used seventeen key words to search for health workforce documents on the Department of Health and Aged Care website (Supporting Information, table 1). We included publicly accessible policy documents concerned only with the planning, governance, or management of the health workforce that were effective until at least 1 January 2024. We did not include inappropriate document types (eg, meeting agendas, books, brochures, campaign certification statements, case definitions, case studies, datasets, digital images, fact sheets, forms, government responses to inquiries, infographics, letters, meeting minutes, posters, presentations, procedures, policy reviews, public interest certificates, reports, statements, terms of reference), clinical practice policy documents, or documents for which the full text was not available. We conducted our searches during 1 June – 31 October 2024.

### Data charting and data analysis

Demographic data, including dates, titles, sources, policy authors, responsible entities, and publication year, were extracted and entered into an Excel (Microsoft) spreadsheet by two authors (SMT, TN) for the first five documents and thereafter by a single author (TN). In the first phase of analysis, the policies were grouped by primary focus, such as specific health profession (eg, nursing, medical doctors), population group (eg, Aboriginal and Torres Strait Islander health care workforce), or location (eg, rural and remote health care workforce). Each document was assigned to one category only; if a document could be assigned to more than one group, two authors determined the category by consensus. For example, the Aged Care On‐Site Pharmacist Program was assigned to the aged care group, not the pharmacist group because its primary focus was strengthening aged care.

In the second phase of analysis, we assessed the focus and scope of each policy using coding. One author undertook the initial coding, and selected documents in each group were reviewed by a second author, followed by a discussion to resolve differences. Each document was coded according to:

- its alignment with one or more strategic domains: supply, distribution, or performance;^20^
- the service sectors affected by the policy: primary care, secondary care, tertiary care, Aboriginal and Torres Strait Islander health care, aged care, mental health care, rural health care, pharmacy, emergency and trauma care; rehabilitation and disability care; public health; or occupational health. Some institutions, such as hospitals, provide services in multiple care sectors, but our categories reflect the dominant service function of each policy rather than an exhaustive classification of all service settings; and
- any substantive mention of specific health professions.

Documents could be coded for more than one strategic domain, sector, or profession.

Finally, we adopted both a directed and conventional content analysis method to analyse the documents by policy instrument types.^21^ As the application of policy type labels (eg, program, scheme, project, initiative) were inconsistently applied, we generated a glossary to ensure consistency in labelling, drawing on definitions used in the policy literature (Box 1). Further, as suggested by Howlett and Ramesh,^19^ we considered how policy instruments include government‐led, mixed, and voluntary instruments, ranging from those that give the state (government) direct control to those with minimal state involvement (Supporting Information, figure 1), and from policy instruments that are mandatory and coercive in nature to those that are voluntary. These features informed an adapted policy hierarchy, as used in the political sciences and policy studies, in which the overarching instruments (often laws) are at the top and the most localised and operational instruments (eg, procedures) at the bottom.^23^, ^24^ In this hierarchy, policies in the lower levels are ideally nested within and aligned with those in higher levels. Based on the glossary and adapted hierarchy, we categorised all documents according to the degree of state (government) involvement, policy instrument type, and degree of mandatory requirements (Supporting Information, figure 2).

Box 1 Adapted glossary of policy document types*

**Table:** 

Policy documents	Definition	Level of government involvement	Policy instrument types	Document types (if relevant)	Mandatory requirement
Law	Formal legal document that outlines rules, regulations enacted by government agencies to implement statutes.	High	Government‐led	Direction document	Mandatory
Agreement	Legally binding contract between two or more parties that outlines their rights and duties.	High	Government‐led	Direction document	Mandatory
Strategy	High level document more focused than policy and outlines course of actions for achieving long term objectives.	High	Government‐led	Direction document	Not mandatory
Plan	Complements a strategy; describes specific steps and actions, objectives, and responsibilities for achieving a strategy.	High	Government‐led	Direction document	Not mandatory
Framework	Structured guide to a concept in a topic area. It outlines detailed principles, roles, and processes to ensure consistency in decision making and policy implementation.	High	Government‐led	Supporting document	Mandatory
Standard	Describes in detail technical elements and criteria to ensure uniformity in a particular topic area.	High	Government‐led	Supporting document	Not mandatory
Guideline	Describes recommended actions for dealing with a question in a particular topic area.	High	Government‐led	Supporting document	Not mandatory
Scheme	Government‐led response to a particular problem; often includes specific eligibility requirements.	Medium	Mixed	NA	Not mandatory
Program	Includes a series of components that are consistently coordinated with detailed implementation plans for achieving broad policy goals.	Medium	Mixed	NA	Not mandatory
Sub‐program	Component or stream of a program. It focuses on a specific aspect of the overall goals of the program and is generally continuous.	Medium	Mixed	NA	Not mandatory
Project	Specific action with a defined timeline (one time event), usually narrowly focused by targeting a small group or one aspect of the problem for achieving a specific goal.	Medium	Mixed	NA	Not mandatory
Incentive	Financial or other benefit provided to an individual to motivate specific behaviour that supports a specific project or structured program.	Medium	Mixed	NA	Not mandatory
Grant	Financial and competitive award to an individual by the government or private foundation to support a specific project or structured program.	Medium	Mixed	NA	Not mandatory

NA = not applicable. * Modified from the Queensland government enterprise architecture.^22^

### Ethics approval

The James Cook University human research ethics committee exempted the study from formal ethics review.

## Results

Of 3380 policy documents initially identified on the Department of Health and Aged Care website (Supporting Information, table 1), 709 were duplicates; we excluded 2452 documents after screening their titles, abstracts, and summaries, including 2026 that were inappropriate document types, 400 not focused on health workforce policy, and 26 outside the time scope of our analysis. We assessed the full text of 219 documents; 98 documents were excluded because they included insufficient information about national health workforce policy. We therefore included 121 policy documents in our analysis (Supporting Information, figure 3).

Using definitions adapted from the policy literature, we identified misclassifications in at least 44 policy documents (Supporting Information, table 2), such as the Health Workforce Scholarship Program (a grant mechanism) and the First Nations Health Worker Traineeship Program (a sub‐program of the Indigenous Australians’ Health Program).

We defined ten main policy groups: four by specific health care profession (nurses and midwives, medical doctors and specialists, allied health care, pharmacists); three by ethnic background (Aboriginal and Torres Strait Islander health care workforce), geographic location (rural health care workforce) or career development (medical and health students or trainees); two by specific areas of health care (aged care health workforce, mental health care workforce); and one for the general health care workforce (Supporting Information, table 3).

By policy group, the number of documents was greatest for the rural health workforce (35), aged care (22), and Aboriginal and Torres Strait Islander health workforce (19); the numbers were lowest for pharmacy (three) and allied health (one), and none had public health or emergency care as their focus (Box 2; Supporting Information, tables 4 to 13).

Box 2 Federal health workforce policy documents: major groups by primary focus*

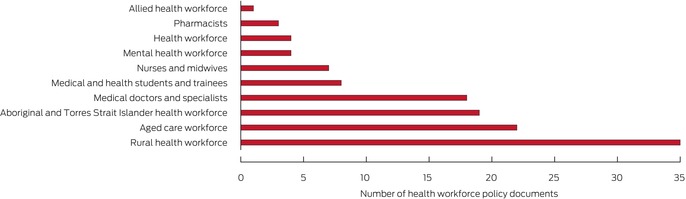

* Each document was assigned to one group only, according to the focus indicated by its title. The data underlying this graph are included in the Supporting Information, table 2; details for the individual documents are included in the Supporting Information, tables 4 to 13.

In most categories, time‐limited programs, sub‐programs, and grants were the most frequent policy types; the age range of policy documents within policy groups spanned up to 20 years (Supporting Information, tables 4 to 13). For the rural health care workforce group, twelve of 35 documents concerned grants and twelve programs; one agreement, one strategy, and no plans were identified. For the aged care workforce group, nine of 22 documents concerned programs and three sub‐programs and incentives; one agreement, one strategy, and no plans were identified. For the Aboriginal and Torres Strait Islander health care workforce group, seven of 19 documents concerned programs, four sub‐programs, and three grants; no legislation or agreements were identified. For the medical doctors and specialists group, 13 of 18 documents concerned programs; no strategies or plans were identified. Mixed policy instruments (multiple interest group programs, sub‐programs, incentives, grants) were more numerous (98 documents) than government‐led instruments (23 documents) (Box 3).

Box 3 Federal health workforce policy documents: by major group and policy type*

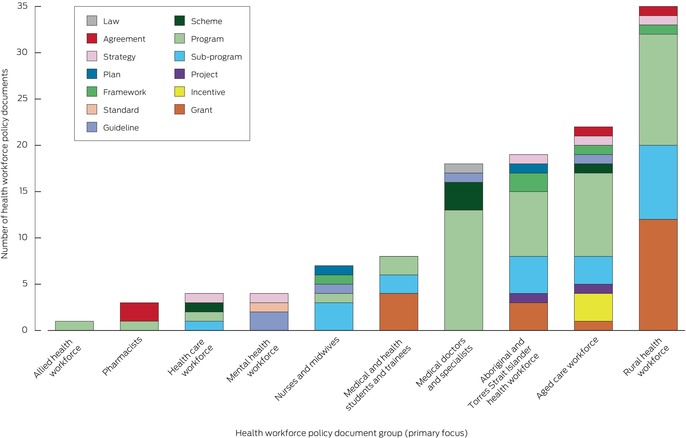

* Each document was assigned to one group only, according to the focus indicated by its title, and one policy type only (definitions: Box 1). The data underlying this graph are included in the Supporting Information, table 14.

Medical doctors and specialists or nurses and midwives were the primary focus of 108 of 121 policy documents. No health workforce documents explicitly referred to medical laboratory scientists, one referred to paramedics, and nine referred to dentists and dental practitioners. Allied health professionals were mentioned in 29 policy documents (Box 4), but the allied health workforce was the primary focus of only one document. Occupational health and rehabilitation and disability were not mentioned in any documents, and few concerned public health (one document) or emergency and trauma care (two documents). The primary care health workforce, a key federal government policy domain, was the subject of 24 documents (Supporting Information, figure 4). The federal Department of Health and Aged Care was the primary author of 106 of the 121 policy documents; twelve other policy authors from several sectors were also identified (Box 5).

Box 4 Federal health workforce policy documents that explicitly referred to specific health professional types*

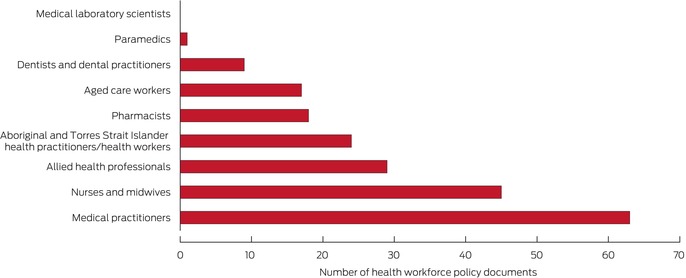

* Documents could be coded to include references to more than one profession. The data underlying this graph are included in the Supporting Information, table 15.

Box 5 **Federal health workforce policy documents: policy authors**

**Table:** 

Issuing entity	Documents
**Governmental entities**	
Department of Health and Aged Care*	106
Department of Education*	3
National Aboriginal and Torres Strait Islander Health Standing Committee (Australian Health Ministers’ Advisory Council)	1
Department of Foreign Affairs and Trade	1
Department of Home Affairs	1
Department of Social Services	1
Chief Nursing and Midwifery Officers Australia	1
Council of Australian Government	1
Office of National Rural Health Commissioner	1
**Statutory bodies**	
Australian Health Practitioner Regulation Agency and National Boards	1
**Professional associations**	
Australian Indigenous Doctors’ Association	1
Services for Australian Rural and Remote Allied Health	1
**Non‐government organisations/advocacy groups**	
Australian Rotary Health	3

* Includes one policy co‐issued by the two departments.

The strategic domain of health workforce supply was a focus of 72 documents, performance of 57 documents, and distribution of 42 documents. Within the domain of health workforce supply, 24 documents were related to time‐bound programs and 19 to grants (Box 6).

Box 6 Federal health workforce policy documents: by strategic policy domains and policy type*

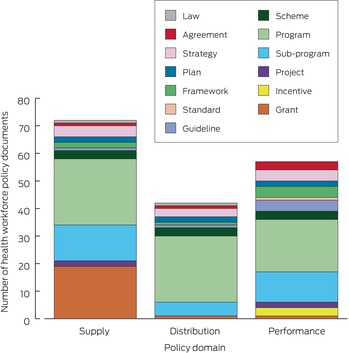

* Each document was assigned to more than one strategic policy domain. The data underlying this graph are included in the  Supporting Information, table 17.

## Discussion

Our analysis highlights key aspects of federal health workforce policy in Australia, including its fragmentation, inconsistent focus on specific professions and service sectors, and a high volume of short term, supply‐driven workforce interventions using mixed instruments.

A major concern that emerges from our review is the highly fragmented nature of health workforce policy at the national level. Separate workforce strategies, programs, and sub‐programs operate concurrently across professional groups and service sectors. Among professions, these include the National Medical Workforce Strategy^25^ and the Nurse Practitioner Workforce Plan,^26^ and service sector‐specific policies include the Stronger Rural Health Strategy,^27^ the National Mental Health Workforce Strategy,^28^ and the National Aboriginal and Torres Strait Islander Health Workforce Strategic Framework and Implementation Plan.^29^

This fragmentation is compounded by the age range of the policy documents (some categories include policies that span 15–20 years) and by the prevalence of short term (if renewable) interventions using mixed instruments. Such interventions can facilitate collaboration between government and non‐government bodies and expedite their effect but also increase the complexity of coordination and alignment. Further, long term evaluation of mixed instrument interventions is made difficult by weaker and often more expensive accountability mechanisms.^30^

In this fragmented policy environment, supply‐focused interventions have become the dominant if limited mechanism for workforce development. The reliance on training and incentive‐based strategies in federal health workforce policy reflects, in part, the attempt to overcome shortages in a system in which coordinated, long term planning is difficult.^31^ However, supply‐focused strategies are often insufficient for strengthening the health workforce in the long term if they do not take labour market dynamics into account, such as employment conditions, retention policies, and alignment with cross‐sectoral strategies, including those of aged care and disability care.^32^

The interaction between policy fragmentation and the dominance of supply‐focused interventions highlights a weakness in the current approach to workforce planning in Australia. Rather than a strategic, future‐oriented policy framework focused on workforce preparedness, isolated policies respond to immediate and profession‐specific workforce gaps.^33^ Unresolved are a series of longstanding structural and workforce readiness problems — the geographic distribution of the workforce, building multidisciplinary team‐based care, balancing specialist disciplines, reducing reliance on overseas‐trained doctors, adaptability and surge capacity for meeting public health emergencies — as well as those in new areas such as digital health, artificial intelligence (AI), and precision medicine.^34^ Meeting these challenges requires a shift from short term interventions that respond to profession‐specific shortages towards integrated, future‐oriented strategies in which consideration of dynamic, multi‐level drivers — such as demographic change, evolving models of care, jurisdictional funding and governance arrangements, education and training pathways, and broader labour market forces — is integrated into a high level framework capable of guiding (without unduly constraining) jurisdictional approaches.^35^ The United Kingdom,^36^ the United States,^37^ and New Zealand^38^ have used this overarching strategic approach.

The lack of a cohesive, long term workforce strategy is not simply a policy deficit but a structural outcome of the governance landscape in which workforce planning is embedded,^1^, ^13^, ^14^, ^15^ a decentralised system that distributes responsibility across multiple levels and bodies. While the federal government finances primary care, public health, aged care, mental health care, and Aboriginal and Torres Strait Islander health care, most health workers are employed by state and territory governments and private sector organisations.^39^ Further, the federal government funds a range of non‐government and statutory bodies involved in workforce policy and planning (eg, peak Aboriginal Community Controlled Health Organisation bodies, primary health networks), which are key commissioners of primary care and rural health workforce agencies. Multiple bodies operating in parallel inevitably complicates policy alignment.^40^ Coordination and policy alignment difficulties were further exacerbated by the 2014 disbandment of Health Workforce Australia, which removed the only national mechanism — albeit then still in development — for workforce policy integration.^2^ Without a national mechanism to support policy integration, the fragmentation we have described is not simply a persistent problem but the predictable outcome of a highly decentralised and structurally disjointed health workforce governance system.

A second major finding of our review was the lack of consistency in policy nomenclature, even in policies authored by a single federal authority, the Department of Health and Ageing. Although perhaps individually unimportant, inconsistent terminology hinders identification of older policies, obscures links between initiatives, and makes their implementation more difficult. This is especially important given the more than 20‐year age range of active policies included in our review. Standardised nomenclature, such as that we have proposed, could improve clarity and coordination.

Our findings have implications for policy, advocacy, and research. Policymakers need a national, whole‐of‐system approach to workforce planning that reduces fragmentation and improves coordination across the federal, state and territory, and private sectors. Re‐establishing a coordinating body like the former Health Workforce Australia, although not a quick solution, could establish a governance mechanism that supports long term, cross‐jurisdictional planning. Researchers must move beyond single profession analyses to examine whose interests are currently shaping policies and how fragmented workforce policies are shaping population health. Advancing these priorities could support more strategic and responsive policy and consequently a more effective health workforce capable of meeting the complex health system needs of Australia in the coming decades.

### Limitations

We analysed policy content but not formulation, implementation, or impact. Policy volume may reflect government focus but does not indicate coherence or effectiveness. We restricted our analysis to policies issued by the Department of Health and Aged Care, but immigration policies issued directly by the Department of Home Affairs play a role in workforce availability. Our review is limited by its federal focus, as most health workforce policy in Australia is the purview of state and territory governments. We are currently undertaking complementary research in this area.

## Conclusion

Our findings indicate the extent of federal health workforce policy fragmentation, which has persisted over time and reflects the governance complexities of the Australian health system. Our findings could inform future research, discussion, and advocacy. Our review is one component of a broader investigation of the structural challenges of workforce policymaking in Australia, with the aim of informing a more coherent, coordinated approach.

## Open access

Open access publishing facilitated by James Cook University, as part of the Wiley – James Cook University agreement via the Council of Australian University Librarians.

## Competing interests

No relevant disclosures.

## Data sharing

The study data can be accessed by contacting the corresponding author.

## Author contributions

Conceptualisation: Stephanie M Topp; methodology: Stephanie M Topp, Thu Nguyen; data curation: Thu Nguyen; formal analysis: Stephanie M Topp, Thu Nguyen, Lana M Elliott; writing (original draft): Stephanie M Topp, Thu Nguyen; writing (review and editing): Stephanie M Topp, Thu Nguyen, Lana M Elliott; funding acquisition: Stephanie M Topp; supervision: Stephanie M Topp.

Received 9 December 2024, accepted 7 May 2025

## Supporting information


**Data S1** Supplementary Figures and Tables
