# Supplementary material for: Federal health workforce policy in Australia and its implications: a descriptive policy document review
Source: Med J Aust. 2025 Aug 17;223(9):459–66. doi: 10.5694/mja2.70021 (PMC12579920; doi:10.5694/mja2.70021)
Supplement: Supplementary file 1 — Data S1 Supplementary Figures and Tables [file MJA2-223-459-s001.pdf]

MJA
The Medical Journal of Australia

Supporting Information

Supplementary methods and results

This appendix was part of the submitted manuscript and has been peer reviewed.
It is posted as supplied by the authors.

Appendix to: Topp SM, Nguyen T, Elliott LM. Federal health workforce policy in Australia and its implications: a descriptive policy document review. *Med J Aust* 2025; doi: 10.5694/mja2.70021.

Figure 1. Adapted spectrum of policy instruments*

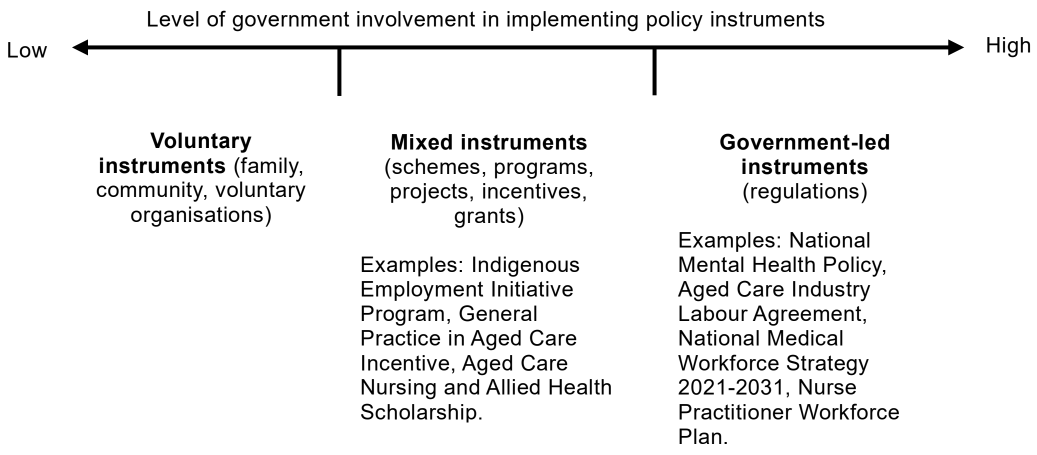

* Adapted from: 1) Howlett M. Managing the "hollow state": procedural policy instruments and modern governance. Canadian Public Administration 2008; 43: 412-431; and 2) Howlett M, Ramesh M, Perl A. Studying public policy, policy cycles and policy subsystems. 2nd edition. Ontario: Oxford University Press, 2003.

Figure 2. Australian federal health workforce policy instrument framework

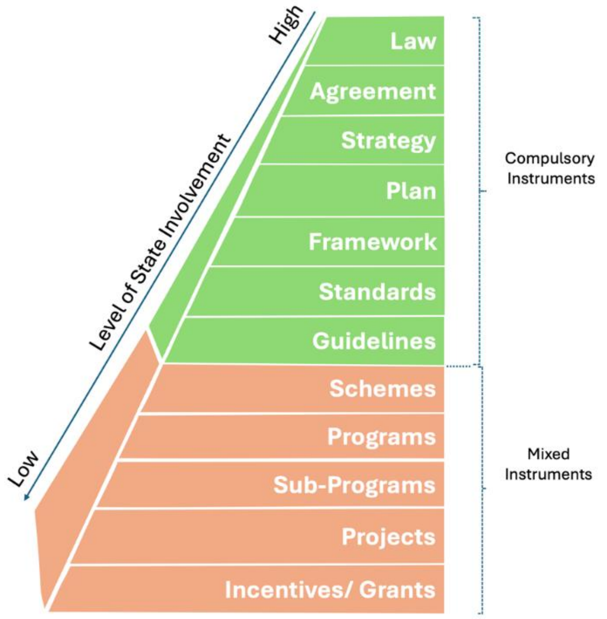

Table 1. Search terms and results for health workforce policy documents from the website of the Australian Department of Health and Aged Care*

	Number of documents, by website tab			
**Search terms**	**Topic**	**Our work**	**Publications**	**Total**
Aboriginal and Torres Strait Islander health workforce	46	30	19	95
Aged care workforce	156	48	280	484
Mental health workforce	46	18	53	117
Rural health workforce	245	58	59	362
Workforce	0	91	546	637
Medical doctors and specialist	173	16	4	193
Nurses and midwives	65	12	8	85
Allied health professionals	115	35	25	175
Aboriginal and Torres Strait Islander health practitioners	0	21	5	26
Aboriginal and Torres Strait Islander health workers	0	26	21	47
Aboriginal and Torres Strait Islander health	28	72	374	474
Dentists and dental practitioners	441	2	1	444
Medical and health students and trainees	98	5	0	103
Pharmacy	26	14	63	103
Pharmacists	0	16	12	28
Paramedics	0	3	3	6
Medical laboratory scientists	0	0	1	1
			TOTAL	3380

* The terms “primary health care” and “general practice” were not used because they would yield policies unrelated to health workforce. We instead identified relevant primary care workforce policies in targeted searches using “health workforce” with related terms.

Figure 3. Policy document search and selection*

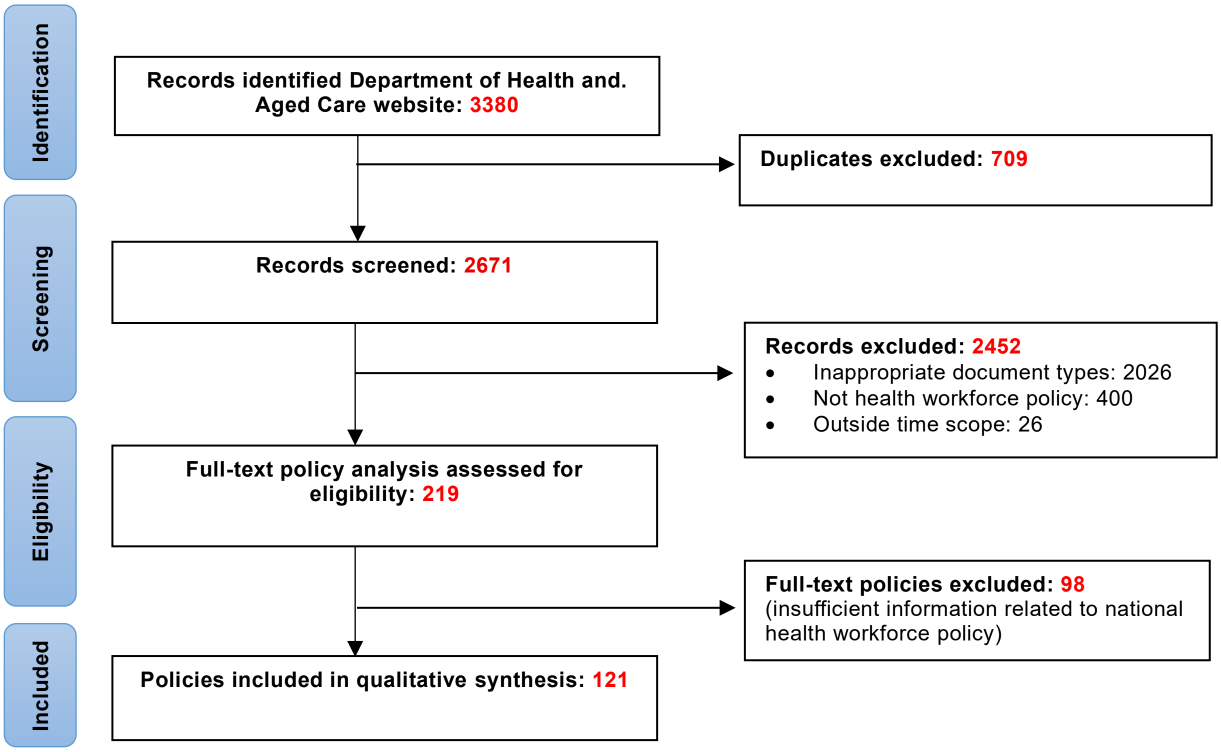

Table 2. Federal health workforce policy documents with inconsistent policy nomenclature

No	Named policy documents organised by major groupings	Policy type as labelled by Department of Health and Aged Care	Policy type as labelled by authors of MJA review
	**Aboriginal and Torres Strait Islander health workforce**		
1	First Nations Health Worker Traineeship Program	Program	Sub-program
2	Indigenous Program of Experience in the Palliative Approach (IPEPA)	Program	Sub-program
3	Practice Incentives Program	Program	Sub-program
4	Remote Area Health Corps	Program	Sub-program
5	Aboriginal and Torres Strait Islander Pharmacy Assistant Traineeship Scheme	Scheme	Grant
6	Aboriginal and Torres Strait Islander Pharmacy Scholarship Scheme (ATIPSS)	Scheme	Grant
	**Aged care workforce**		
7	Aged care on-site pharmacists	Measure	Program
8	Program of Experience in the Palliative Approach (PEPA) in Aged Care	Program	Sub-program
9	Rural, Remote and First Nations Aged Care Service Development Assistance Panel (SDAP)	Program	Sub-program
10	Rural and Remote Aged Care Management Training Program	Program	Sub-program
11	Aged Care Registered Nurses' Payment to reward clinical skills and leadership	Payment	Incentive
	**Allied health workforce**		
12	The Allied Health Rural Generalist (AHRG) Pathway	Pathway	Program
	**Health Workforce**		
13	Program of Experience in the Palliative Approach (PEPA)	Program	Sub-program
	**Nurses and midwives**		
14	Transition to Practice Program	Program	Sub-program
15	Building Nurses Capacity Program	Program	Sub-program
16	Chronic Disease Management and Healthy Aging (CDMHA)	Program	Sub-program
	**Medical and health students and trainees**		
17	Rural Health Multidisciplinary Training Program	Program	Sub-program
18	Palliative Care curriculum for Undergraduates (PCC4U)	Program	Sub-program
19	Nursing and Allied Health Scholarship	Program	Grant
20	Primary Care Nursing and Midwifery Scholarship Program	Program	Grant
21	Grants and Scholarships for Dental Students	Scholarships	Grant
	**Medical doctors and specialists**		
22	Amendments to the Higher Education Support Act 2003 – Debtor Guidelines	Act	Law
23	Single Employer Model (SEM) Trials	Trials	Scheme
	**Mental health workforce**		
	**Pharmacists**		
	**Rural health workforce**		
24	The Ngayyubah Gadan Consensus Statement – Rural and Remote Multidisciplinary Health Teams	Consensus statement	Framework
25	The Murray-Darling Medical Schools Network	Network	Program
26	The National Rural Generalist Pathway	Pathway	Program
27	Streamlining General Practice Training	Initiative	Program
28	Rural Generalist Training Scheme	Scheme	Sub-program
29	The John Flynn Prevocational Doctor Program	Program	Sub-program

No	Named policy documents organised by major groupings	Policy type as labelled by Department of Health and Aged Care	Policy type as labelled by authors of MJA review
30	Workforce Incentive Program – Practice Stream	Program	Sub-program
31	Workforce incentive Stream – Doctor Stream	Program	Sub-program
32	Workforce Incentive Stream – Rural Advanced Skill	Program	Sub-program
33	Australian Rotary Health Rural Medical Scholarships	Scholarship	Grant
34	Australian Rotary Health Rural Nursing Scholarship	Scholarship	Grant
35	Administrative Support to Pharmacy Schools Scheme	Scheme	Grant
36	Health Workforce Scholarship Program	Program	Grant
37	Intern Incentive Allowance for Rural Pharmacies	Allowance	Grant
38	Rural Continuing Professional Education Allowance	Allowance	Grant
39	Rural Intern Training Allowance	Allowance	Grant
40	Rural Pharmacy Liaison Officer Program	Program	Grant
41	Rural Pharmacy Scholarship Mentor Scheme	Scheme	Grant
42	Rural Pharmacy Scholarship Scheme	Scheme	Grant
43	Rural Pharmacy Student Placement Allowance Program	Program	Grant
44	Rural and Regional Enterprise Scholarships Program	Program	Grant

Table 3. Federal health workforce policy documents: major groups by primary focus

Group	Policy documents
Rural health workforce	35
Aged care workforce	22
Aboriginal and Torres Strait Islander health workforce	19
Medical doctors and specialists	18
Medical and health students and trainees	8
Nurses and midwives	7
Mental health workforce	4
Health workforce	4
Pharmacists	3
Allied health workforce	1

Table 4. Federal health workforce policy documents, coded by policy type, profession, sector, strategic focus, author (coded issuing entity), year of publication: Aboriginal and Torres Strait Islander health workforce

No.	Policy Instrument	Document names	Document types	Coded professions	Coded sectors	Coded policy domains	Coded issuing entities	Publication year
1	Government-led	National Scheme's Aboriginal and Torres Strait Islander Health and Cultural Safety Strategy 2020-2025	Strategy	Aboriginal and Torres Strait Islander health practitioners/health workers	Aboriginal and Torres Strait Islander health	Performance	AHPRA & National Boards	2020
2	Government-led	National Aboriginal and Torres Strait Islander Health Workforce Strategic Framework and Implementation Plan 2021-2031 (new National Workforce Plan)	Plan	Aboriginal and Torres Strait Islander health practitioners/health workers;<br>Allied health professionals;<br>Medical practitioners;<br>Nurses and midwives	Aboriginal and Torres Strait Islander health	Supply;<br>Distribution;<br>Performance	Department of Health and Aged Care	2022
3	Government-led	Aboriginal and Torres Strait Islander Health Curriculum Framework	Framework	Aboriginal and Torres Strait Islander health practitioners/health workers;<br>Allied health professionals;<br>Medical practitioners;<br>Nurses and midwives	Aboriginal and Torres Strait Islander health	Supply	Department of Health and Aged Care	2014
4	Government-led	Cultural Respect Framework 2016-2026 for Aboriginal and Torres Strait Islander Health	Framework	Aboriginal and Torres Strait Islander health practitioners/health workers	Aboriginal and Torres Strait Islander health	Performance	The National Aboriginal and Torres Strait Islander Health Standing Committee (NATSIHSC) of the Australian Health Ministers' Advisory Council	2016
5	Mixed	Indigenous Australian's Health Program (IAHP)	Program	Aboriginal and Torres Strait Islander health practitioners/health workers	Aboriginal and Torres Strait Islander health	Performance	Department of Health and Aged Care	2014
6	Mixed	Australian General Practice Training and Aboriginal and Torres Strait Islander Salary Support Program Policy 2024	Program	Medical practitioners	Aboriginal and Torres Strait Islander health	Supply;<br>Performance	Department of Health and Aged Care	2024

No.	Policy Instrument	Document names	Document types	Coded professions	Coded sectors	Coded policy domains	Coded issuing entities	Publication year
7	Mixed	Australia Indigenous Doctors' Association (AIDA)Specialist Trainee Support Program (STSP)	Program	Medical practitioners	Aboriginal and Torres Strait Islander health	Supply; Distribution	The Australian Indigenous Doctor's Association (AIDA)	2022
8	Mixed	Indigenous Employment Initiative (IEI) Program	Program	Aboriginal and Torres Strait Islander health practitioners/ health workers	Aboriginal and Torres Strait Islander health	Supply; Performance	Department of Health and Aged Care	2020
9	Mixed	Indigenous Health Workforce Traineeships program (IHWT)	Program	Aboriginal and Torres Strait Islander health practitioners/health workers;<br>Allied health professionals;<br>Dentists and dental practitioners;<br>Medical practitioners;<br>Nurses and midwives	Aboriginal and Torres Strait Islander health	Supply	Department of Health and Aged Care	2020
10	Mixed	Medical Outreach Indigenous Chronic Disease Program (MOICDP)	Program	Medical practitioners	Aboriginal and Torres Strait Islander health<br>Rural health	Performance	Department of Health and Aged Care	2015
11	Mixed	Support for Aboriginal and Torres Strait Islander health professional organisations	Program	Aboriginal and Torres Strait Islander health practitioners/ health workers	Aboriginal and Torres Strait Islander health;<br>Rural health	Performance	Department of Health and Aged Care	2022
12	Mixed	First Nations Health Worker Traineeship Program (under the overarching IAHP)	Sub-program	Aboriginal and Torres Strait Islander health practitioners/ health workers	Aboriginal and Torres Strait Islander health	Supply	Department of Health and Aged Care	2022
13	Mixed	Indigenous Program of Experience in the Palliative Approach (IPEPA) (under the overarching Palliative Care Education and Training Collaborative Program)	Sub-program	Allied health professionals;<br>Aboriginal and Torres Strait Islander health practitioners/health workers;<br>Nurses and midwives	Aboriginal and Torres Strait Islander health	Performance	Department of Health and Aged Care	2003

No.	Policy Instrument	Document names	Document types	Coded professions	Coded sectors	Coded policy domains	Coded issuing entities	Publication year
14	Mixed	Practice Incentives Program - Indigenous Health Incentive (under the overarching IAHP)	Sub-program	Aboriginal and Torres Strait Islander health workers/practitioners	Aboriginal and Torres Strait Islander health	Performance	Department of Health and Aged Care	2010
15	Mixed	Remote Area Health Corps (under the overarching IAHP)	Sub-program	Allied health professionals;<br>Dentists and dental practitioners;<br>Medical practitioners;<br>Nurses and midwives	Aboriginal and Torres Strait Islander health;<br>Rural health	Distribution	Department of Health and Aged Care	2008
16	Mixed	Gwandalan National Palliative Care Project	Project	Allied health professionals;<br>Aboriginal and Torres Strait Islander health practitioners/health workers;<br>Medical practitioners;<br>Nurses and midwives	Aboriginal and Torres Strait Islander health	Supply;<br>Performance	Department of Health and Aged Care	2020
17	Mixed	Aboriginal and Torres Strait Islander Pharmacy Assistant Traineeship Scheme (ATSIPATS)	Grant	Pharmacists	Aboriginal and Torres Strait Islander health	Supply;<br>Distribution	Department of Health and Aged Care	2012
18	Mixed	Aboriginal and Torres Strait Islander Pharmacy Scholarship Scheme (ATIPSS)	Grant	Pharmacists	Aboriginal and Torres Strait Islander health	Supply	Department of Health and Aged Care	2011
19	Mixed	Australian Rotary Health Indigenous Health Scholarships	Grant	Medical practitioners;<br>Nurses and midwives;<br>Allied health professionals;<br>Aboriginal and Torres Strait Islander health practitioners/health workers	Aboriginal and Torres Strait Islander health	Supply	Australian Rotary Health	2003

Table 5. Federal health workforce policy documents, coded by policy type, profession, sector, strategic focus, author (coded issuing entity), year of publication: aged care workforce

No.	Policy Instrument	Document names	Document types	Coded professions	Coded sectors	Coded policy domains	Coded issuing entities	Publication year
1	Government-led	The Aged Care Industry Labour Agreement	Agreement	Aged care workers	Aged care	Distribution	Department of Health and Aged Care	2023
2	Government-led	My Aged Care Workforce Learning Strategy 2023	Strategy	Nurses and midwives; Aged care workers	Aged care	Supply	Department of Health and Aged Care	2023
3	Government-led	My Aged Care Quality Learning Framework	Framework	Nurses and midwives; Aged care workers	Aged care	Supply; Performance	Department of Health and Aged Care	2024
4	Government-led	Aged Care Worker Wages - Guidance for aged care providers on the provision of funding relating to Stage 2 of the Fair Work Commission Aged Care Work Value Case	Guideline	Nurses and midwives; Aged care workers	Aged care	Performance	Department of Health and Aged Care	2023
5	Mixed	Pacific Australia Labour Mobility (PALM) Scheme	Scheme	Aged care workers	Aged care	Supply; Distribution	Department of Foreign Affairs and Trade	2023
6	Mixed	Aged Care Capital Assistance Program (ACCAP)	Program	Aged care workers	Aged care	Performance;	Department of Health and Aged Care	2024
7	Mixed	Aged care on-site pharmacist	Program	Pharmacists	Aged care	Distribution	Department of Health and Aged Care	2024
8	Mixed	Aged Care Nursing Clinical Placements Program	Program	Aged care workers	Aged care	Supply	Department of Health and Aged Care	2023
9	Mixed	Aged care Transition to Practice Program	Program	Nurses and midwives	Aged care	Performance	Department of Health and Aged Care	2023
10	Mixed	Boosting the Local Care Workforce Program	Program	Nurses and midwives; Aged care workers	Aged care	Performance	Department of Social Services	2017
11	Mixed	Business, Industry and Regional Outreach (BIRO) program	Program	Aged care workers	Aged care	Distribution	Department of Home Affairs	2020
12	Mixed	Dementia Training Program	Program	Aged care workers	Aged care	Performance	Department of Health and Aged Care	2022
13	Mixed	Home Care Workforce Support Program	Program	Aged care workers	Aged care	Distribution; Performance	Department of Health and Aged Care	2022

No.	Policy Instrument	Document names	Document types	Coded professions	Coded sectors	Coded policy domains	Coded issuing entities	Publication year
14	Mixed	Rural Locum Assistance Program for Aged Care	Program	Nurses and midwives; Aged care workers	Aged care	Distribution	Department of Health and Aged Care	2011
15	Mixed	Program of Experience in the Palliative Approach (PEPA) in Aged Care (under the overarching Palliative Care Education and Training Collaborative)	Sub-program	Nurses and midwives; Aged care workers	Aged care	Supply; Performance	Department of Health and Aged Care	2003
16	Mixed	Rural, Remote and First Nations Aged Care Service Development Assistance Panel (SDAP) (under the overarching Indigenous Australians' Health Program)	Sub-program	Nurses and midwives; Aged care workers	Aged care	Supply; Performance	Department of Health and Aged Care	2011
17	Mixed	Rural and Remote Aged Care Management Training Program (under the Rural, Remote, and First Nations Aged Care Service Development Assistance Panel SDAP)	Sub-program	Aged care workers	Aged care	Supply	Department of Health and Aged Care	2022
18	Mixed	Advance Project	Project	Medical practitioners	Aged care	Supply; Performance	Department of Health and Aged Care	2016
19	Mixed	Aged Care Registered Nurses' Payment to reward clinical skills and leadership	Incentive	Nurses and midwives	Aged care	Performance	Department of Health and Aged Care	2022
20	Mixed	General Practice in Aged Care Incentive	Incentive	Medical practitioners	Aged care	Performance	Department of Health and Aged Care	2024
21	Mixed	Relocation Incentive and Retention Bonuses, (under the overarching Rural LAP)	Incentive	Aged care workers	Aged care	Performance	Department of Health and Aged Care	2022
22	Mixed	Aged Care Nursing and Allied Health Scholarship	Grant	Allied health professionals; Nurses and midwives	Aged care	Supply	Department of Health and Aged Care	2023

Table 6. Federal health workforce policy documents, coded by policy type, profession, sector, strategic focus, author (coded issuing entity), year of publication: allied health workforce

No.	Policy Instrument	Document names	Document types	Coded professions	Coded sectors	Coded policy domains	Coded issuing entities	Publication year
1	Mixed	The Allied Health Rural Generalist (AHRG) Pathway	Program	Allied health professionals	Rural health	Supply; Distribution; Performance	Services for Australian Rural & Remote Allied Health (SARRAH)	2013

Table 7. Federal health workforce policy documents, coded by policy type, profession, sector, strategic focus, author (coded issuing entity), year of publication: general health workforce

No.	Policy Instrument	Document names	Document types	Coded professions	Coded sectors	Coded policy domains	Coded issuing entities	Publication year
1	Government-led	National Medical Workforce Strategy 2021-2031	Strategy	Medical practitioners; Nurses and midwives; Allied health professionals; Aboriginal and Torres Strait Islander health practitioners/ health workers	Primary care; Secondary care; Tertiary care; Aboriginal and Torres Strait Islander health; Aged care; Mental health; Rural health; Public health	Supply; Distribution; Performance	Department of Health and Aged Care	2021
2	Mixed	National Registration and Accreditation Scheme (NRAS)	Scheme	Aboriginal and Torres Strait Islander health practitioners/health workers; Medical practitioners; Allied health professionals; Dentists and dental practitioners; Nurses and midwives; Paramedics; Pharmacists	Primary care; Secondary care; Tertiary care; Aboriginal and Torres Strait Islander health; Aged care; Mental health; Rural health Pharmaceutical Emergency and trauma care	Supply	The Councils of Australian Governments	2010

No.	Policy Instrument	Document names	Document types	Coded professions	Coded sectors	Coded policy domains	Coded issuing entities	Publication year
3	Mixed	Palliative Care Education and Training Collaborative	Program	Aboriginal and Torres Strait Islander health practitioners/health workers; Allied health professionals; Medical practitioners; Nurses and midwives;	Primary care; Aboriginal and Torres Strait Islander health; Aged care;	Supply	Department of Health and Aged Care	2003
4	Mixed	Program of Experience in the Palliative Approach (PEPA) (under the overarching Palliative Care Education and Training Collaborative)	Sub-program	Allied health professionals; Medical practitioners; Nurses and midwives	Primary care	Supply	Department of Health and Aged Care	2003

Table 8. Federal health workforce policy documents, coded by policy type, profession, sector, strategic focus, author (coded issuing entity), year of publication: nurses and midwives

No.	Policy Instrument	Document names	Document types	Coded professions	Coded sectors	Coded policy domains	Coded issuing entities	Publication year
1	Government-led	Nurse Practitioner Workforce Plan	Plan	Nurses and midwives	Primary care; Secondary care; Tertiary care; Aboriginal and Torres Strait Islander health; Mental health; Aged care; Rural health	Supply; Distribution; Performance	Department of Health and Aged Care	2023
2	Government-led	National Rural and Remote Nursing Generalist Framework 2023-2027	Framework	Nurses and midwives	Rural health	Distribution; Performance	The National Rural Health Commissioner	2023

No.	Policy Instrument	Document names	Document types	Coded professions	Coded sectors	Coded policy domains	Coded issuing entities	Publication year
3	Government-led	Advanced Nursing Practice-Guidelines for the Australian Context	Guideline	Nurses and midwives	Primary care;<br>Secondary care;<br>Tertiary care;<br>Aboriginal and Torres Strait Islander health;<br>Mental health;<br>Aged care;<br>Rural health	Performance	The Chief Nursing and Midwifery Officers Australia	2020
4	Mixed	Strengthening the role of the nursing workforce (sometimes are referred to as " Nurses in Primary Health Care Program")	Program	Nurses and midwives	Rural health	Supply;<br>Distribution;<br>Performance	Department of Health and Aged Care	2015
5	Mixed	Transition to Practice Program (under the overarching Stronger Rural Health Strategy)	Sub-program	Nurses and midwives	Rural health	Supply;<br>Performance	Department of Health and Aged Care	2015
6	Mixed	Building Nurses capacity Program (under the overarching Stronger Rural Health Strategy)	Sub-program	Nurses and midwives	Rural health	Performance	Department of Health and Aged Care	2015
7	Mixed	Chronic Disease Management and Healthy Aging (CDMHA) (under the overarching Stronger Rural Health Strategy)	Sub-program	Nurses and midwives	Rural health	Supply;<br>Performance	Department of Health and Aged Care	2015

Table 9. Federal health workforce policy documents, coded by policy type, profession, sector, strategic focus, author (coded issuing entity), year of publication: medical and health students and trainees

No.	Policy Instrument	Document names	Document types	Coded professions	Coded sectors	Coded policy domains	Coded issuing entities	Publication year
1	Mixed	Australian General Practice Training (AGPT) Program	Program	Medical practitioners	Primary care	Supply; Distribution	Australian Government Department of Health and Aged Care	2023
2	Mixed	Exchange Visitor Sponsorship Program (EVSP)	Program	Medical practitioners	Primary care; Secondary care; Tertiary care; Aboriginal and Torres Strait Islander health; Aged care; Mental health; Rural health	Supply	Department of Health and Aged Care	2023
3	Mixed	Rural Health Multidisciplinary Training Program (under the overarching Indigenous Australians' Health Program, and under the Stronger Rural Health Strategy)	Sub-program	Allied health professionals; Dentists and dental practitioners; Medical practitioners; Nurses and midwives	Rural health	Supply; Distribution	Department of Health and Aged Care	2016
4	Mixed	Palliative curriculum for undergraduates (PCC4U) (under the overarching Palliative Care Education and Training Collaborative Program)	Sub-program	Aboriginal and Torres Strait Islander health practitioners/health workers; Allied health professionals; Medical practitioners; Nurses and midwives	Primary care; Aboriginal and Torres Strait Islander care; Aged care;	Supply	Department of Health and Aged Care	2003
5	Mixed	Nursing and Allied Health scholarships	Grant	Nurses and midwives; Allied health professionals	Primary care; Secondary care; Tertiary care; Aboriginal and Torres Strait Islander health; Aged care; Mental health; Rural health	Supply	Department of Health and Aged Care	2023

No.	Policy Instrument	Document names	Document types	Coded professions	Coded sectors	Coded policy domains	Coded issuing entities	Publication year
6	Mixed	Primary Care Nursing and Midwifery Scholarship Program	Grant	Nurses and midwives	Primary care;<br>Secondary care;<br>Tertiary care;<br>Aged care;<br>Aboriginal and Torres Strait Islander health;<br>Rural health;<br>Mental health	Supply	Department of Health and Aged Care	2023
7	Mixed	Higher Education Loan Program (HELP)	Grant	Aboriginal and Torres Strait Islander health practitioners/health workers;<br>Allied health professionals;<br>Medical practitioners;<br>Nurses and midwives;<br>Dentists and dental practitioners	Primary care;<br>Secondary care;<br>Tertiary care;<br>Aged care;<br>Aboriginal and Torres Strait Islander health;<br>Rural health;<br>Mental health	Supply	Department of Education	2005
8	Mixed	Grants and scholarships for dental students	Grant	Dentists and dental practitioners	Primary care;<br>Aged care;<br>Aboriginal and Torres Strait Islander health;<br>Rural health	Supply	Department of Health and Aged Care	2021

Table 10. Federal health workforce policy documents, coded by policy type, profession, sector, strategic focus, author (coded issuing entity), year of publication: medical doctors and specialists

No.	Policy Instrument	Document names	Document types	Coded professions	Coded sectors	Coded policy domains	Coded issuing entities	Publication year
1	Government-led	Amendments to the Higher Education Support Act 2003 - HELP Debtor guidelines (Health Practitioners) 2023	Law	Medical practitioners; Nurses and midwives	Primary care; Secondary care; Tertiary care; Aged care; Aboriginal and Torres Strait Islander health; Rural health; Mental health	Supply; Distribution	Department of Education & Department of Health and Aged Care	2023
2	Government-led	General Practice Fellowship Program Placement Guidelines	Guideline	Medical practitioners	Primary care	Supply	Department of Health and Aged Care	2019
3	Mixed	Five Year International Medical Graduates Recruitment Scheme, formerly known as the Five Year Overseas Trained Doctors Scheme	Scheme	Medical practitioners	Primary care; Secondary care; Tertiary care; Aboriginal and Torres Strait Islander health; Aged care; Mental health; Rural health	Supply; Distribution; Performance	Department of Health and Aged Care	2023
4	Mixed	Medical and midwife professional indemnity insurance schemes	Scheme	Allied health professionals; Medical practitioners; Nurses and midwives	Primary care; Secondary care; Tertiary care; Aboriginal and Torres Strait Islander health; Aged care; Mental health; Rural health	Performance	Department of Health and Aged Care	2002
5	Mixed	Single Employer Model (SEM) Trial	Scheme	Medical practitioners	Primary care	Distribution; Performance	Department of Health and Aged Care	2023

No.	Policy Instrument	Document names	Document types	Coded professions	Coded sectors	Coded policy domains	Coded issuing entities	Publication year
6	Mixed	Approved Medical Deputising Services (AMDS) Program	Program	Medical practitioners	Primary care; Rural health	Performance	Department of Health and Aged Care	2021
7	Mixed	Approved Private Emergency Department (APED) Program	Program	Medical practitioners	Tertiary care; Emergency and trauma care	Supply	Department of Health and Aged Care	2019
8	Mixed	Advanced Skills Training Posts Rural Generalists and General Practitioners Program (AST Program)	Program	Medical practitioners	Rural health	Supply	Department of Health and Aged Care	2024
9	Mixed	Flexible Approach to Training in Expanded Settings (FATES)	Program	Medical practitioners	Secondary care; Tertiary care; Rural health	Supply; Distribution	Department of Health and Aged Care	2021
10	Mixed	General Practitioner Procedural Training Support Program (GPPTSP)	Program	Medical practitioners	Primary care	Supply	Department of Health and Aged Care	2010
11	Mixed	Other Medical Practitioners Programs	Program	Medical practitioners	Primary care; Secondary care; Tertiary care; Rural health	Distribution	Department of Health and Aged Care	2004
12	Mixed	Practice Incentives program (PIP)	Program	Medical practitioners	Primary care	Performance	Department of Health and Aged Care	2019
13	Mixed	Pre-Fellowship Program (PFP)	Program	Medical practitioners	Primary care; Rural health	Distribution	Department of Health and Aged Care	2014
14	Mixed	Remote Vocational Training Scheme (through Remote Vocational Training Scheme Ltd)	Program	Medical practitioners	Rural health	Supply	Department of Health and Aged Care	2000
15	Mixed	Special Approved Placements Program (SAPP)	Program	Medical practitioners	Primary care; Rural health	Supply	Department of Health and Aged Care	2003
16	Mixed	Specialist Training Program	Program	Medical practitioners	Secondary care; Tertiary care; Rural health	Supply; Distribution	Department of Health and Aged Care	2022
17	Mixed	Supporting Rural Specialists in Australia	Program	Medical practitioners	Secondary care; Tertiary care; Rural health	Supply; Performance	Department of Health and Aged Care	2017

No.	Policy Instrument	Document names	Document types	Coded professions	Coded sectors	Coded policy domains	Coded issuing entities	Publication year
18	Mixed	Temporary Resident Other Medical Practitioners Program	Program	Medical practitioners	Tertiary care; Rural health	Performance	Department of Health and Aged Care	2001

Table 11. Federal health workforce policy documents, coded by policy type, profession, sector, strategic focus, author (coded issuing entity), year of publication: mental health workforce

No.	Policy Instrument	Document names	Document types	Coded professions	Coded sectors	Coded policy domains	Coded issuing entities	Publication year
1	Government-led	National Mental Health Workforce Strategy 2022-2032	Strategy	Allied health professionals; Medical practitioners; Nurses and midwives	Mental health	Supply; Distribution; Performance	Department of Health and Aged Care	2022
2	Government-led	National Practice Standards for the Mental Health Workforce 2013	Standard	Allied health professionals; Nurses and midwives	Mental health	Performance	Department of Health and Aged Care	2013
3	Government-led	Primary Health Networks (PHN) Mental Health Care Guidance - Workforce Support Information and Resources	Guideline	Allied health professionals	Mental health	Performance	Department of Health and Aged Care	2017
4	Government-led	Primary Health Networks (PHN) Mental Health Care Guidance - Peer Workforce Role in Mental Health and Suicide Prevention	Guideline	Allied health professionals	Mental health	Distribution; Performance	Department of Health and Aged Care	2019

Table 12. Federal health workforce policy documents, coded by policy type, profession, sector, strategic focus, author (coded issuing entity), year of publication: pharmacists

No.	Policy Instrument	Document names	Document types	Coded professions	Coded sectors	Coded policy domains	Coded issuing entities	Publication year
1	Government-led	Eight Community Pharmacy Agreement (8CPA)	Agreement	Pharmacists	Pharmaceutical	Performance	Department of Health and Aged Care	2024
2	Government-led	Strategic Agreement on Pharmacist Professional Practice	Agreement	Pharmacists	Pharmaceutical	Performance	Department of Health and Aged Care	2024
3	Mixed	Pharmacy Trial Program	Program	Pharmacists	Pharmaceutical	Distribution	Department of Health and Aged Care	2017

Table 13. Federal health workforce policy documents, coded by policy type, profession, sector, strategic focus, author (coded issuing entity), year of publication: rural health workforce

No.	Policy Instrument	Document names	Document types	Coded professions	Coded sectors	Coded policy domains	Coded issuing entities	Publication year
1	Government-led	Landmark Compact with the RACGP "Strengthening Medicare: Agreement with the Australian College of General Practitioners (RACGP) on behalf of General Practitioners"	Agreement	Medical practitioners	Rural health	Supply; Performance	Department of Health and Aged Care	2017
2	Government-led	The Stronger Rural Health Strategy	Strategy	Medical practitioners; Nurses and midwives	Rural health	Supply; Distribution; Performance	Department of Health and Aged Care	2018
3	Government-led	The Ngayyubah Gadan Consensus Statement - Rural and Remote Multidisciplinary Health Teams	Framework	Medical practitioners; Nurses and midwives; Allied health professionals; Aboriginal and Torres Strait Islander health practitioners/ health workers	Rural health	Performance	Department of Health and Aged Care	2023
4	Mixed	The Murray-Darling Medical Schools Networks	Program	Medical practitioners	Rural health	Supply; Distribution	Department of Health and Aged Care	2021
5	Mixed	National Rural Generalist Pathway	Program	Medical practitioners	Rural health	Supply	Department of Health and Aged Care	2019

No.	Policy Instrument	Document names	Document types	Coded professions	Coded sectors	Coded policy domains	Coded issuing entities	Publication year
6	Mixed	Junior Doctor Training Program (JDTP)	Program	Medical practitioners	Rural health	Supply; Distribution	Department of Health and Aged Care	2018
7	Mixed	Streamlining General Practice Training	Program	Medical practitioners	Rural health	Supply; Distribution	Department of Health and Aged Care	2019
8	Mixed	Bonded Medical Program (replacing Bonded Medical Places Scheme)	Program	Medical practitioners	Rural health	Distribution	Department of Health and Aged Care	2020
9	Mixed	Royal Flying Doctor Service Program	Program	Allied health professionals; Dentists; Medical practitioners; Nurses and midwives	Rural health	Distribution	Department of Health and Aged Care	1930s
10	Mixed	Visas for GPs Targeting Areas of Doctors Shortages Program	Program	Medical practitioners	Rural health	Supply; Distribution	Department of Health and Aged Care	2019
11	Mixed	Workforce Incentive Program (WIP)	Program	Medical practitioners	Rural health	Supply; Distribution; Performance	Department of Health and Aged Care	2020
12	Mixed	Emergency Locum Service Program (ELS)	Program	Pharmacists	Rural health	Distribution	Department of Health and Aged Care	2002
13	Mixed	Innovative Models of Care (IMOC) Program	Program	Medical practitioners; Nurses and midwives; Allied health professionals; Aboriginal and Torres Strait Islander health practitioners/health workers; Pharmacists	Rural health	Distribution	Department of Health and Aged Care	2020
14	Mixed	Rural Procedural Grants Program	Program	Medical practitioners	Rural health	Distribution; Performance	Department of Health and Aged Care	2004
15	Mixed	Rural Health Workforce Support Activity	Program	Medical practitioners	Rural health	Distribution; Performance	Department of Health and Aged Care	2017
16	Mixed	Junior Doctor Training - Private Hospital Stream	Sub-program	Medical practitioners	Rural health	Supply; Distribution	Department of Health and Aged Care	2018
17	Mixed	Junior Doctor Training - Rural Primary Care Stream	Sub-program	Medical practitioners	Rural health	Supply; Distribution	Department of Health and Aged Care	2018

No.	Policy Instrument	Document names	Document types	Coded professions	Coded sectors	Coded policy domains	Coded issuing entities	Publication year
18	Mixed	Rural Generalist Training Scheme (under the overarching National rural Generalist Pathway)	Sub-program	Medical practitioners	Rural health	Supply	Department of Health and Aged Care	2021
19	Mixed	The John Flynn Prevocational Doctor Program (JFPDP) (a component of the National Rural Generalist Pathway)	Sub-program	Medical practitioners	Rural health	Distribution	Department of Health and Aged Care	2023
20	Mixed	Workforce Incentive Program (WIP) - Practice Stream, replaced the Practice Nurse Incentive Program	Sub-program	Allied health professionals; Aboriginal and Torres Strait Islander health practitioners/health workers; Nurses and midwives	Rural health; Aboriginal and Torres Strait Islander health	Performance	Department of Health and Aged Care	2020
21	Mixed	Workforce Incentive Program - Doctor Stream, replaced the General Practice Incentive Program	Sub-program	Medical practitioners	Rural health	Performance	Department of Health and Aged Care	2020
22	Mixed	Workforce Incentive Program - Rural Advanced Skill (under the overarching National Rural Generalist Pathway)	Sub-program	Medical practitioners	Rural health	Performance	Department of Health and Aged Care	2023
23	Mixed	Rural Health Continuing Education (RHCE)	Sub-program	Allied health professionals; Medical practitioners; Aboriginal & Torres Strait Islander health practitioners/health workers	Rural health	Supply; Performance	Department of Health and Aged Care	2010
24	Mixed	Australian Rotary Health Rural Medical Scholarships	Grant	Medical practitioners	Rural health	Supply	Australian Rotary Health	2007
25	Mixed	Australian Rotary Health Rural Nursing Scholarships	Grant	Nurses and midwives	Rural health	Supply	Australian Rotary Health	2007
26	Mixed	Administrative Support to pharmacy Schools Scheme	Grant	Pharmacists	Rural health	Supply	Department of Health and Aged Care	2015

No.	Policy Instrument	Document names	Document types	Coded professions	Coded sectors	Coded policy domains	Coded issuing entities	Publication year
27	Mixed	Health Workforce Scholarship Program	Grant	Medical practitioners;<br>Nurses and midwives;<br>Allied health professionals;<br>Dentists and dental practitioners;<br>Aboriginal and Torres Strait Islander health practitioners/health workers	Rural health;<br>Aboriginal and Torres Strait Islander health	Performance	Department of Health and Aged Care	2022
28	Mixed	Intern Incentive Allowance for Rural Pharmacies	Grant	Pharmacists	Rural health<br>Pharmaceutical	Supply	Department of Health and Aged Care	2015
29	Mixed	Rural Continuing Professional Education Allowance	Grant	Pharmacists	Rural health<br>Pharmaceutical	Supply	Department of Health and Aged Care	2015
30	Mixed	Rural Intern Training Allowance	Grant	Pharmacists	Rural health;<br>Pharmaceutical	Supply	Department of Health and Aged Care	2015
31	Mixed	Rural Pharmacy Liaison Officer Program	Grant	Pharmacists	Rural health;<br>Pharmaceutical	Supply	Department of Health and Aged Care	2015
32	Mixed	Rural Pharmacy Scholarship Mentor Scheme	Grant	Pharmacists	Rural health;<br>Pharmaceutical	Supply	Department of Health and Aged Care	2015
33	Mixed	Rural Pharmacy Scholarship Scheme	Grant	Pharmacists	Rural health;<br>Pharmaceutical	Supply	Department of Health and Aged Care	2015
34	Mixed	Rural Pharmacy Student Placement Allowance program	Grant	Pharmacists	Rural health;<br>Pharmaceutical	Supply	Department of Health and Aged Care	2015
35	Mixed	Rural and Regional Enterprise Scholarships Program	Grant	Allied health professionals;<br>Dentists dental practitioners;<br>Medical practitioners;<br>Nurses and midwives;<br>Aboriginal and Torres Strait Islander health practitioners/health workers;<br>Pharmacists;<br>Aged care workers	Rural health	Supply	Department of Education	2016

Table 14. Federal health workforce policy documents: by major group and policy type

Major group	Grant	Incentive	Project	Sub-program	Program	Scheme	Guideline	Standard	Framework	Plan	Strategy	Agreement	Law
Allied health workforce	0	0	0	0	1	0	0	0	0	0	0	0	0
Pharmacists	0	0	0	0	1	0	0	0	0	0	0	2	0
Health workforce	0	0	0	1	1	1	0	0	0	0	1	0	0
Mental health workforce	0	0	0	0	0	0	2	1	0	0	1	0	0
Nurses and midwives	0	0	0	3	1	0	1	0	1	1	0	0	0
Medical and health students and trainees	4	0	0	2	2	0	0	0	0	0	0	0	0
Medical doctors and specialists	0	0	0	0	13	3	1	0	0	0	0	0	1
Aboriginal and Torres Strait Islander health workforce	3	0	1	4	7	0	0	0	2	1	1	0	0
Aged care workforce	1	3	1	3	9	1	1	0	1	0	1	1	0
Rural health workforce	12	0	0	8	12	0	0	0	1	0	1	1	0

Table 15. Federal health workforce policy documents that explicitly refer to specific health professional types

Professional type	Number of policy documents
Medical practitioners	63
Nurses and midwives	45
Allied health professionals	29
Aboriginal and Torres Strait Islander health practitioners/health workers	24
Pharmacists	18
Aged care workers	17
Dentists and dental practitioners	9
Paramedics	1
Medical laboratory scientists	0

Figure 4. Service sectors to which federal health workforce policy documents refer*

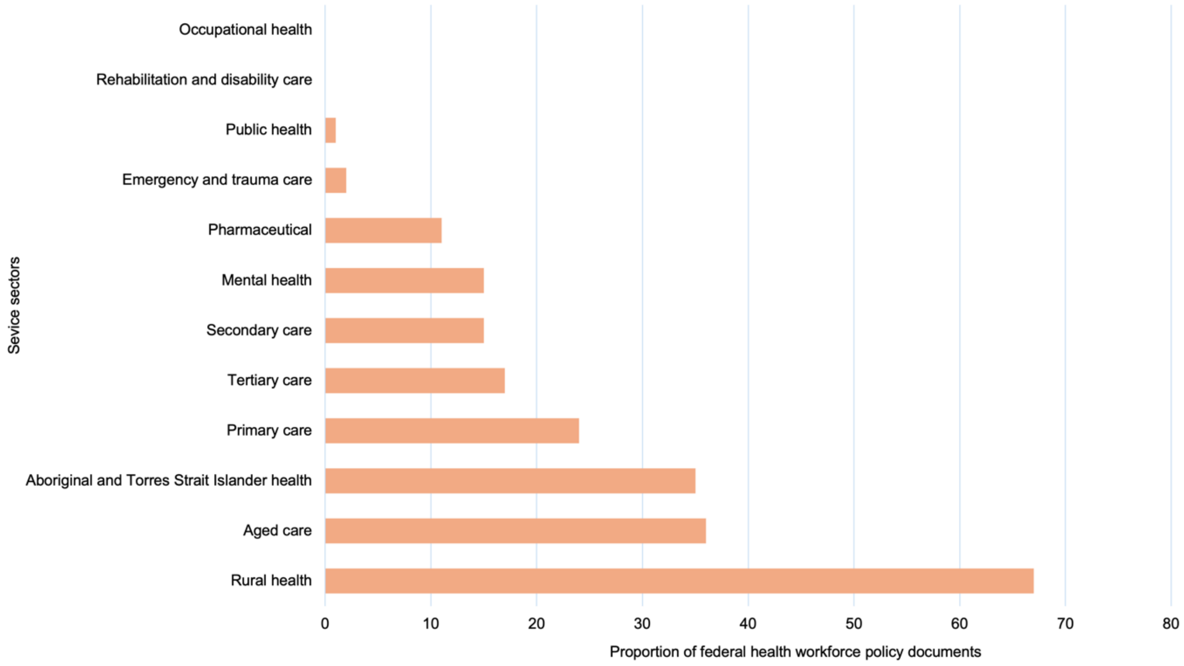

* A service sub-sector could be referenced directly or indirectly in the document; coding was by consensus. Documents could be coded to more than one service sector. The data underlying this graph are included in table 16.

Table 16. Service sectors to which federal health workforce policy documents refer

Sectors	Number of policy documents
Rural health	67
Aged care	36
Aboriginal and Torres Strait Islander health	35
Primary care	24
Tertiary care	17
Secondary care	15
Mental health	15
Pharmacy	11
Emergency and trauma care	2
Public health	1
Rehabilitation and disability care	0
Occupational health	0

Table 17. Federal health workforce policy documents: by strategic policy domains and policy type

Policy type	Supply	Distribution	Performance
Grant	19	1	1
Incentive	0	0	3
Project	2	0	2
Sub-program	13	5	11
Program	24	24	19
Scheme	3	3	3
Guideline	1	1	4
Standard	0	0	1
Framework	2	1	4
Plan	2	2	2
Strategy	4	3	4
Agreement	1	1	3
Law	1	1	0
